# Multiple Biological Activities of *Rhododendron przewalskii* Maxim. Extracts and UPLC-ESI-Q-TOF/MS Characterization of Their Phytochemical Composition

**DOI:** 10.3389/fphar.2021.599778

**Published:** 2021-02-10

**Authors:** Lixia Dai, Jian He, Xiaolou Miao, Xiao Guo, Xiaofei Shang, Weiwei Wang, Bing Li, Yu Wang, Hu Pan, Jiyu Zhang

**Affiliations:** ^1^ Key Laboratory of New Animal Drug Project, Gansu Province, Key Laboratory of Veterinary Pharmaceutical Development of Ministry of Agriculture, Lanzhou Institute of Husbandry and Pharmaceutical Sciences, Chinese Academy of Agricultural Sciences, Lanzhou, China; ^2^ Tibetan Medical College of Qinghai University, Xining, China; ^3^ State Key Laboratory of Tibetan Medicine Research and Development, Xining, China

**Keywords:** flavonoids, antifungal activity, anti-inflammatory, antioxidant activity, *Rhododendron* przewalskii maxim

## Abstract

**Backgroud:** *Rhododendron* przewalskii Maxim. is an evergreen shrub that is used as a traditional medicine in China. However, the modern pharmacology and the chemical components of this plant has not been studied. In this paper, we aimed to investigate the antifungal, anti-inflammatory and antioxidant activities and underlying mechanism of its aqueous and ethanol extracts, and analyze their chemical composition and active compounds of *R. przewalskii*.

**Methods:** The antifungal activity was determined *in vitro*, and anti-inflammatory and antioxidant activities and underlying mechanism of its aqueous and ethanol extracts were evaluated *in vitro* and in RAW 264.7 cells. The chemical composition were analyzed using UPLC-ESI-Q-TOF/MS, and the contents of six compounds were determined via HPLC.

**Results:** Both extracts of *R. przewalskii* showed promising anti-inflammatory activity *in vitro*; decreased the production of four inflammatory cytokines, namely, nitric oxide, IL-1β, IL-6 and TNF-*ɑ*, in RAW 264.7 cells induced by lipopolysaccharide; and exhibited weak cytotoxicity. The extracts significantly scavenged DPPH radicals, superoxide radicals and hydroxyl radicals to exert antioxidant effects *in vitro*. The two extracts also exhibited cellular antioxidant activity by increasing superoxide dismutase and CAT activities and decreasing malondialdehyde content in RAW 264.7 cells induced by LPS. However, the antifungal activity of the two extracts was weak. Nine flavonoids were identified by UPLC-ESI-Q-TOF/MS. Of these, six compounds were analyzed quantitatively, including avicularin, quercetin, azaleatin, astragalin and kaempferol, and five compounds (myricetin 3-O-galactoside, paeoniflorin, astragalin, azaleatin and kaempferol) were found in this species for the first time. These compounds demonstrated antioxidant activities that were similar to those of the *R. przewalskii* extracts and were thought to be the active compounds in the extracts*.*

**Conclusion:** *R. przewalskii* extracts presented promising anti-inflammatory and antioxidant activities. The extracts contained amounts of valuable flavonoids (8.98 mg/g fresh material) that were likely the active compounds in the extract contributing to the potential antioxidant activity. These results highlight the potential of *R. przewalskii* as a source of natural antioxidant and anti-inflammatory agents for the pharmaceutical industry.

## Introduction

*Rhododendron* L., one of the largest genera in the family Ericaceae, comprises eight subgenera with more than 960 species (Qiang et al., 2011). Most of the species are distributed in Southeast Asia and the Himalayan region of the Northern Hemisphere, while others grow in Europe, North America and northeastern Australia (Qiang et al., 2011; Guo et al., 2017). The bioactivity of this genus has been investigated, and many plants have been demonstrated to have significant biological activities, including nitric oxide (NO)-production inhibitory activity, anti-HIV activity, histamine-release inhibitory activity, tyrosinase inhibitory activity, anti-diabetic activity, anti-inflammatory and antinociceptive activities, cytotoxicity, antioxidant activity and other activities (Qiang et al., 2011; Mok et al, 2013; Rapinski et al., 2016). In addition, hundreds of secondary metabolites have been isolated from this genus, mainly flavonoids and diterpenoids (Qiang et al., 2011). Traditionally, medicinal plants have been used for centuries by different civilizations as therapeutic agents due to their preventive and curative properties (Tasneem et al., 2019).

*Rhododendron przewalskii* Maxim. is an evergreen shrub that grows widely at altitudes of 2,900–4,300 masl on the Qinghai-Tibet Plateau in northwestern China, including in Gansu, Sichuan, Qinghai and Shanxi provinces (Yang et al., 2017). For thousands of years, the flowers and leaves of this plant have been widely used as a folk medicine to treat lung diseases, inflammation and general body weakness (Chinese materia editorial committee, 2002). The flowers of *R. przewalskii,* as well as the aerial parts of *Morina kokonorica*, the roots of *Aconitum kusnezoffii* and *Przewalskia tangutica* were used to heal wound and inflammation; the flowers and leaves together with the rhizomes of *Acorus calamus* and musk have the anti-inflammatory and antiparasitic activities and were widely used in clinic by local people (Chinese materia editorial committee, 2002). In a previous field investigation, we found that *R. przewalskii* was widely used by local people to treat upper respiratory diseases and pneumonia in humans and animals (Shang et al., 2012). The chemical composition of this species, which includes compounds such as rhododendrone, rhododendronside, rhododendrone A, hyperosides (1*R*,3*R*,6*S*)-1,5,5-trimethyl-6-[(1*E*)-3-oxobut-1-en-1-yl]-7-oxabicyclo [4.1.0]hept-3-yl-β-d-glucopyranoside, grayanotoxin I, pieroside A, ursolic acid (+)-catechin (β)-rhododendrol, and pinoresinol 4-O-β-d-glucopyranoside, has been reported in only four studies (Li and Jia, 2003; Jia and Li, 1996; Liang et al., 2014, 2016). However, to date, the biological activity of this plant and its extract has not been screened and studied to our knowledgement.

Because of the divide between the widespread use of *R. przewalskii* in folk medicine and the limited research on this plant, there is a growing interest in identifying more active compounds and finding the significant pharmacological activities of *R. przewalskii* for possible uses in the nutraceutical and medicinal industries. In this paper, we first studied the antifungal, anti-inflammatory and antioxidant activities of an aqueous extract and ethanol extract of *R. przewalskii* and elucidated its possible mechanism of action. Then, the phytochemical compositions of the extracts were analyzed by UPLC-ESI-Q-TOF/MS, and the antioxidant activities of the six identified compounds were demonstrated. The objectives of this study were to determine the biological activities of *R. przewalskii* and its active compounds for potential pharmaceutical industry uses.

## Materials and Methods

### Materials and Plants

*Rhododendron przewalskii* Maxim. was collected from the northern slope of a mountain near the Zhuaxixiulong region of Tianzhu (N 37°11.4′, E 102°46.1′, 2,922 masl), Gansu Province, China, in Jul. 2019. It was authenticated by Chaoying Luo, a professor at the Lanzhou Institute of Husbandry and Pharmaceutics Sciences. A voucher specimen with accession number ZSY422 was submitted to the Herbarium of the Lanzhou Institute of Husbandry and Pharmaceutics Sciences, Chinese Academy of Agricultural Sciences (Lanzhou, China).

### Chemicals and Reagents

Hyperoside (98%), astragalin (98%), avicularin (98%), azaleatin (98%), quercetin (98%), dexamethasone (DEX) and kaempferol (98%) were purchased from Shanghai Macklin Inc (Shanghai, China); paeoniflorin (98%) was purchased from Shanghai Acmec Biochemical Co. Ltd. (Shanghai, China) (+)-catechin (95%) and vanillin (95%) was purchased from Shanghai Yuanye Biochemical Co.. Ltd. (Shanghai, China); 1,1-diphenyl-2-picrylhydrazyl (DPPH), lipopolysaccharide (LPS), nitroblue tetrazolium (NBT), nicotinamide adenine dinucleotide disodium salt (NADH-2Na), and phenazine methosulfate (PMS) were purchased from Sigma-Aldrich (St. Louis, MO, United States); and ethylenediaminetetraacetic acid (EDTA), ascorbic acid (Vc), ferrous sulfate (FeSO_4_), salicylate, ferrous chloride (FeCl_2_), hydrogen peroxide (H_2_O_2_), petroleum ether, ethyl acetate, trichloroacetic acid (TCA), thiobarbituric acid (TBA) and acetic acid were purchased from Sinopharm Chemical Reagent Co. (Shanghai, China). Ethanol (analytical grade) was purchased from Tianjin Guangfu Chemical Reagent Company (Tianjin, China); ketoconazole and azoxystrobin were purchased from Solarbio Co. Ltd (Beijing, China); and acetonitrile (HPLC and MS grade) was purchased from Fisher Scientific (England).

### Crude Extract Preparation

The aqueous extract of *R. przewalskii* (AERP) was prepared as follows. Four hundred grams of the raw fresh leaves and branches was decocted at 100 °C with 3,000 ml water three times for 1 h each time. Then, the decoction was filtered and dried, and the aqueous extract was obtained (Mahomoodally et al., 2020).

The ethanol extract of *R. przewalskii* (EERP) was prepared as follows. Four hundred grams of fresh raw material was placed in 75% ethanol solution for 2 days at the room temperature, and then the solution was filtered and obtained. The residue material was heated with 400 ml of 75% ethanol solution to reflux three times for 1 h each time. After extraction, all solutions were combined, filtered and dried, and the ethanol extract was obtained (Liang et al., 2014; Liu et al., 2018b).

### Antifungal Activity

The fungi *Aspergillus niger* (ATCC 9642), *Candida albicans* (ATCC 10231) and *Saccharomyces cerevisiae* (ATCC 7753) were obtained from the American Type Culture Collection (ATCC). According to described methods (Obistioiu et al., 2014; Liu et al., 2018a; Alonso-Esteban et al., 2019), the antifungal activities of the AERP and EERP at 5 mg/ml and 2 mg/ml were tested by measuring the diameter of clear inhibition zone. Briefly, a volume of 100 μL of fungi suspensions (1 × 10^6^ CFU/ml) were streaked on the surface of Sabouraud Dextrose Agar (SDA, Solarbio, China) for fungal cultures. Then, the oxford cup (5 mm) containing 200 µL of extracts was placed on the surface of seeded Petri plates. After incubating for 24 h at 30°C, The inhibition zones were evaluated by measuring the diameter of clear inhibition zone around the oxford cup using a vernier caliper which was recorded as an indication of antifungal activity. Ketoconazole (0.01–0.05 mg/ml) was used as a positive control. Experiments were performed in triplicate.

The plant pathogenic fungi *Botrytis cinerea,* Magnaporthe oryzae*, Penicillium cyclopium, Rhizopus stolonifer*, *Rhizoctonia solani* and *Sclerotinia sclerotiorum* were obtained from the Agricultural Culture Collection of China. According to a previously described method (Shang et al., 2019), the anti-fungal activities of the two extracts at 5 mg/ml and 2 mg/ml were determined. Briefly, Petri plates (90 mm), containing potato dextrose agar (PDA, Solarbio, China) were inoculated with 5 mm plugs of mycelia. Extracts were dissolved in DMSO and then added to the PDA to obtain the two concentrations (5 mg/ml and 2 mg/ml, respectively). When the fungal growth in the control had completely covered the dishes, the diameters of the mycelia in treatment groups were measured. Five replicates were performed. Azoxystrobin (0.1 mg/ml) was used as a positive control. Experiments were performed in triplicate. The inhibition percentages were calculated by using Eq. 1.

$$\text{Inhibition rate}\left(\%\right)=\left[\frac{\left(\text{C}-\text{d}\right)-\left(\text{S}-\text{d}\right)}{\text{C}-\text{d}}\right]\times 100\%,$$ (1)

where C and S represent the average diameters of the fungal colony of control and sample, respectively, and d is diameter of the fungal cakes.

## Anti-Inflammatory Assay

### Cell Culture and Cell Viability

The primary mouse macrophage RAW 264.7 cell line was obtained from Prof. Zhang’s lab., Lanzhou Institute of Husbandry and Pharmaceutical Sciences, CAAS (Lanzhou, China), and cultured in culture medium prepared with 10% FBS and 90% DMEM under a humidified incubator of 5% CO_2_ at 37 °C.

The cytotoxicity of the extracts to the RAW 264.7 cells were evaluated through a 2-(2-methoxy-4-nitrophenyl)-3-(4-nitrophenyl)-5-(2,4-disulfophenyl)-2H-tetrazolium and monosodium salt (WST-8) using the ZETA cell counting kit (ZETA Life, United States) (Song et al., 2020). Briefly, RAW 264.7 cells at a density of 5×10^4^ cells/well (100 μL) were incubated in 96-well plates for 24 h, and 10 μL of the AERP and EERP (25–200 μg/ml) were added to each well and incubated again for 24 h. Then, CCK agent (10 μL) were added and cultured continuously for 30 min, and the absorbance of each well was measured at a wavelength of 450 nm using a Multiskan Go Microplate Spectrophotometer (Thermo Scientific., United States). DMSO (0.1%) was used as a control, and three replicates were performed. The inhibitory effect of the two extracts on the growth of the RAW 264.7 cells was calculated by using Eq. 2:

$$\text{Inhibition rate}\left(\%\right)=\left[\frac{\text{ODc}-\text{ODe}}{\text{ODc}}\right]\times 100\%,$$ (2)

where ODc represents the optical density of the control and ODe represents the optical density of the extracts.

### Measurement of Inflammatory Cytokines

According to a previously described method (Vieira et al., 2019), the inhibitory effects of the two extracts on the production of four inflammatory cytokines induced by LPS were investigated. RAW 264.7 cells (500 μL) at a density of 5×10^4^ cells/well were incubated in 48 well plates for 24 h, and the cell supernatants were discarded. Then, 500 μL of the AERP and EERP (25–200 μg/ml) were added and cultured for 1 h; dexamethasone (DEX) (10 and 25 μg/ml) was used as a positive control. Afterwards, LPS (1 μg/ml) was added to each well, and RAW 264.7 cells were stimulated for 24 h to cause inflammation. Finally, the cell culture supernatants were collected to determine the levels of four inflammatory cytokines, nitric oxide (NO), interleukin-1β (IL-1β), IL-6 and tumor necrosis factor-*ɑ* (TNF-*ɑ*)*,* using enzyme-linked immunosorbent assay kits from Nanjingjiancheng Bio (NJJCBIO, China) (Tian et al., 2019). The concentrations of NO (Eq.3), IL-1β (Eq.4), IL-6 (Eq.5) and TNF-*ɑ* (Eq.6) were calculated by the different standard curve equations, and the absorbance of each well was measured at a wavelength of 450 nm using a Multiskan Go Microplate Spectrophotometer (Thermo Scientific., United States). Three replicates were performed.

$$NO\text{content}\left(\mu M\right)=\frac{SP_{OD}-B_{OD}}{ST_{OD}-B_{OD}}\times ST_{\text{concentration}}÷P_{\text{concentration}},$$ (3)

Where *SP*_*OD*_ represents the absorbance of sample, *B*_*OD*_ represents the absorbance of black well, *ST*_*OD*_ represents the absorbance of standard substance, *ST*concentration represents the concentration of standard substance, *P*concentration represents the concentration of protein;

$$Y=2.287/\left[1+{\left(X/5464.333\right)}^{0.503}\right]+1.735.$$ (4)

Equation 4 is used to determine the IL-1β content (ng/L); Where *Y* represents the absorbance of sample, *X* represents the concentration of sample or standard substance;

$$Y=34.408/\left[1+{\left(X/41424.458\right)}^{0.706}\right]-31.406.$$ (5)

Equation 5 is used to determine the IL-6 content (ng/L); Where *Y* represents the absorbance of sample, *X* represents the concentration of sample or standard substance

$$Y=12.557/\left[1+{\left(X/2271.377\right)}^{0.983}\right]-8.5922.$$ (6)

Equation 6 is used to determine the TNF-*ɑ* content (ng/L); Where *Y* represents the absorbance of sample, *X* represents the concentration of sample or standard substance;

### *In vitro* Antioxidant Activity

The *in vitro* antioxidant activities of the two extracts and commercial compounds were determined by studying the radical scavenging effects on DPPH radicals, superoxide radicals, and hydroxyl radicals. The reducing power capacity and metal chelating activity were also determined.

### DPPH Radical Scavenging Activity Assay

According to a previously described method (Brand-Williams et al., 1995), the DPPH radical scavenging activity of the two extracts and compounds was evaluated. At concentrations of 5–1,000 μg/ml, extracts and compounds (100 μL) were added to 96 well plates and incubated with a methanol solution of DPPH (100 μL, 0.2 mM) at 37 °C for 30 min in the dark. The absorbance was recorded at 517 nm using a Multiskan Go Microplate Spectrophotometer (Thermo Scientific., United States). Distilled water and ascorbic acid (Vc, 2.5–10 μg/ml) were used as a negative control and positive control, respectively. Three replicates were performed.

### Superoxide Radical Scavenging Activity Assay

According to a previously described method (Yen and Chen 1995), the superoxide radical scavenging activity of the two extracts and the compounds was evaluated. In addition to the above test, the extracts and compounds (100 μL, 5–1,000 μg/ml, respectively) were placed in 96-well plates and incubated with 100 μL of NADH-2Na (557 µM), 50 μL of PMS (45 µM) and 50 μL of NBT (108 µM) at 25 °C for 5 min, and their absorbance was measured at 510 nm using a Multiskan Go Microplate Spectrophotometer (Thermo Scientific., United States). Distilled water was used as a negative control, and Vc (20–100 μg/ml) was used as a positive control for comparison. Three replicates were performed.

### Hydroxyl Radical Scavenging Activity Assay

According to a previously described method (Yen and Chen 1995), the hydroxyl radical scavenging activity of the two extracts and compounds was evaluated. The extracts and compounds (50 μL, 5–1,000 μg/ml, respectively) were placed in 96-well plates and incubated with 50 μL of FeSO_4_ (9 mM), 50 μL of ethanol-salicylate (9 mM) and H_2_O_2_ (3.8 mM) at 37 °C for 30 min, and the absorbance was measured at 510 nm using a Multiskan Go Microplate Spectrophotometer (Thermo Scientific., United States). Distilled water was used as a negative control, and Vc (20–600 μg/ml) was used as a positive control for comparison. Three replicates were performed.

### Fe^2+^ Chelating Activity

According to a previously described method (Benzie and Strain 1996), the Fe^2+^ chelating activity of the two extracts and compounds was evaluated. Briefly, 100 μL of the two extracts and compounds (5–1,000 μg/ml) and EDTA-2Na (100 μL) were mixed with ferrous chloride (FeCl_2_) solution (5 μL and 2 mM) and ferrozine solution (20 μL and 5 mM) and then left to react for 10 min at room temperature. After adding 75 μL of distilled water, the absorbance was determined at 560 nm using a Multiskan Go Microplate Spectrophotometer (Thermo Scientific., United States). Distilled water was used as a negative control, and EDTA-2Na (20–60 μg/ml) was used as a positive control for comparison. Three replicates were performed.

### Reducing Power

According to a previously described method (Oyaizu 1986), the hydroxyl radical scavenging activity of the two extracts and compounds was evaluated. An amount of 100 μL of the extracts and compounds was added a tube and incubated with 250 μL of sodium phosphate buffer (pH 6.6) and 250 μL of 1% potassium ferrocyanide for 20 min at 50°C. Afterward, 10% trichloroacetic acid solution (TCA, 250 μL) was added and then centrifuged at 4,000 rpm for 10 min. The supernatants (50 μL) were taken and placed into a 96-well plate, and 50 μL of distilled water and 50 μL of ferric chloride were added and mixed. Finally, the absorbance was measured at 700 nm using a Multiskan Go Microplate Spectrophotometer (Thermo Scientific., United States). Distilled water was used as a negative control, and Vc (20–60 μg/ml) was used as a positive control for comparison. Three replicates were performed.

### Cellular Antioxidant Activity

The antioxidant activities were investigated by determining the SOD (superoxide dismutase) and CAT (catalase) activities and MDA (malondialdehyde) contents of the RAW 264.7 cells according to the previously described methods (Chou et al., 2013; Wang et al., 2015; Zhang et al., 2021). RAW 264.7 cells (500 μL) at a density of 5×10^4^ cells/well were incubated in 48-well plates for 24 h, and the cell supernatants were discarded. 500 μL of the AERP and EERP (10–500 μg/ml) were added and cultured for 1 h, and DEX (10 and 25 μg/ml) was used as a positive control. LPS was used to stimulate RAW 264.7 cells for 24 h. Then, the cell supernatants were discarded again, and the cell were digested with trypsin and certrifuged at 8,000 g for 10 min at 4 °C, the cell were harvested. After adding into cell lysis buffer, the samples were ultrasonically lysed 30 times (3 s each) on ice with and centrifuged at 8,000 g for 10 min at 4 °C again. Finally, the supernatants were collected to determine the SOD and CAT activities and MDA content using the relative assay kits (Solarbio, China). The protein concentrations of each samples were determined using BCA kit (Solarbio, China). Briefly, SOD activity in the supernatant was measured by using nitro blue tetrazolium as a substrate with SOD assay kit. After adding reagents according to the kit to supernatant and then incubating at 37 °C for 40 min, color developing agent was added and kept for 10 min. The absorbance was measured at 560 nm using a Multiskan Go Microplate Spectrophotometer (Thermo Scientific., United States). One unit of SOD activity was defined as the amount of enzyme that inhibited autooxidation by 50% under the given experimental condition and the values were expressed as U/mg prot. CAT activity was determined by using H_2_O_2_ as a substrate with CAT assay kit, and the absorbance was scanned at 240 nm with a Multiskan Go Microplate Spectrophotometer (Thermo Scientific., United States). The concentration of MDA, a reliable marker of lipid peroxidation, was measured using a Multiskan Go Microplate Spectrophotometer at 450, 532 and 600 nm. Three replicates were performed.

### Chemical Analysis of *R. przewalskii* Extract

#### UPLC-ESI-Q-TOF/MS Analysis

The analysis was performed on an Agilent Technologies apparatus (1,290 Infinity II, Agilent, United States) containing a quadrupole time-of-flight mass analyzer (QTOF). A ZORBAX Eclipse Plus C_18_ RRHD column (2.1 × 150 mm, 1.8 μm) was used for separation in this assay. The solvent system was composed of 0.1% formic acid solution (A) and acetonitrile (B), and a gradient elution method was applied as follows: 0–35 min 98%–60% A; 35–37 min 60%–10% A; 37–39 min 10% A; 39–41 min 10%–98% A; 41–43 min 98% A with a flow rate of 0.3 ml/min. The total run time was 43 min. EERP (10 mg) were dissolved in methanol and filtered through a 0.22 μm filter, and the filtered solution was used as the final solution for UPLC-ESI-Q-TOF/MS analysis. Mass data acquisition was performed using an Agilent 6530 Q-TOF (Agilent Technologies, United States) equipped with a dual electrospray ionization source (ESI) operating in negative and positive ion modes. The scan time was set at two spectra/s, and the data were collected in centroid mode from 50 to 500 m/z. The desolvation gas rate was set to 10 L/min at 340 °C, and the nebulizer pressure was 45 psi; the fragment voltage was 135 V, and the capillary voltage was 3.5 KV. Raw MS spectra were analyzed by Mass Hunter Qualitative Analysis software (Agilent Technologies, United States).

#### Quantification of Phytochemicals via UPLC

The quantification of the main metabolites accumulated in *R. przewalskii* extracts was performed by UPLC (ultra-performance liquid chromatography). To obtain better separation performance for the qualitative determination, the UPLC condition was optimized according to the previously described UPLC-ESI-Q-TOF/MS method, and the analysis was performed on an Agilent Technologies apparatus (1,290 Infinity II, Agilent, United States). The solvent system was composed of 0.1% formic acid solution (A) and acetonitrile (B). A gradient elution method was applied as follows: 0–5 min 95%–75% A; 5–40 min 75%–30% A; 40–45 min 30%–10% A; 45–46 min 10%–95% A; 46–48 min 95% A with a flow rate of 0.3 ml/min. The total run time was 48 min. The UV detector was set to 356 nm. A symmetry reversed-phase column (C_18_, 4.6 mm × 150 mm; particle size 5 μm, Waters, Ireland) was maintained at ambient temperature (30.0 °C). The mobile phase was filtered through a Millipore 0.45 mm filter and degassed prior to use. EERP (5 mg) were dissolved in methanol (10 ml) and filtered through a 0.22 μm filter, and the filtered solution was used as the final solution for quantitative analysis of the contents of the five compounds using HPLC. The quantification was based on the retention times and UV spectra of commercial compounds. Five milligrams of six standards were weighed and dissolved in 10 ml of methanol, respectively, from which 1 ml was taken and made up to 5 ml with solvent as this stock solution to inject. The equation of the curve and the coefficient of determination were calculated (Barrientos et al., 2020). Three replicates were performed.

#### Qualitative Analysis of Two Compounds via Thin Layer Chromatography (TLC)

For the determination of paeoniflorin, EERP (2.5 g) were dissolved in 95% ethanol and spotted on the silica gel plate (Qindao Haiyang Chemical Reagent Factory, China) with 2 μL, and methylbenzene-ethyl acetate-methanol-water-acetic acid (10:7:5: 0.5:0.1) was used as the developing solvent to develop it. 5% Vanillin sulfuric acid with 10% sulphuric acid ethanol solvent was used as the color developing reagent, and commercial paeoniflorin (98%) was used as standard preparation (Lu et al., 2019).

For the determination of catechin, EERP (2.5 g) were dissolved in 10 ml ethyl acetate, and then were treated by ultrasonic method for 25 min. Subsequently, the solution were filtered, dried, dissolved in methanol and spotted on the silica gel plate with 10 μL, and petroleum ether-ethyl acetate (3:1) was used as the developing solvent to develop it. 5% Vanillin sulfuric acid with 10% sulphuric acid ethanol solvent was used as the color developing reagent and commercial catechin (95%) was used as standard preparation (Liu, 2010).

#### Statistical Analysis

The data obtained were analyzed using SPSS software version 18.0 and expressed as the mean ± S.E.M. Data were analyzed by one-way ANOVA, followed by Dunnett's test when the data involved three or more groups.

## Results

### Extraction Yields and Antifungal Activity

In this paper, we first prepared an aqueous extract and an ethanol extract of *R. przewalskii*, and the yields were 26% and 24%, respectively. Subsequently, the antifungal activity of the two extracts against nine fungal strains, including *A. niger*, *C. albicans*, *S. cerevisiae*, *B. cinerea,* M. oryzae*, P. cyclopium, R. stolonifer*, *R. solani* and *S. sclerotiorum,* was assessed. As shown in Table 1, at concentrations of 5 mg/ml and 2 mg/ml, the extracts did not show any inhibitory effects against *A. niger*, *S. cerevisiae* or *C. albicans* (Supplementary Table S1)*.* This result indicated that the main chemical compounds of the extracts may haven`t the antifungal acitvity, and this species could not be used to treat the diseases infected by above fungi. The toxicities against five plant pathogenic fungi were also weak, and only the AERP showed moderate antifungal activity against *S. sclerotiorum,* with inhibition rates of 67.04% and 47.11% at 5 mg/ml and 2 mg/ml, respectively.

**TABLE 1:** Antifungal activities of two extracts of *R. przewalskii*.

Fungi	AERP (inhibition rates %)		EERP (inhibition rates %)		Ketoconazole (MIC mg/mL)	Azoxystrobin (inhibition rates %)
	5 mg/ml	2 mg/ml	5 mg/ml	2 mg/ml		0.1 mg/ml
*A. niger*	ND^1^	ND	ND	ND	0.025	–
*S. cerevisiae*	ND	ND	ND	ND	0.013	–
*C. albicans*	ND	ND	ND	ND	0.025	–
*B. cinerea*	9.99 ± 1.25^aa^	2.40 ± 0.78^aa^	4.40 ± 1.11^aa^	ND	–	59.51 ± 1.34
M. oryzae	24.94 ± 2.43^aa^	11.12 ± 1.11^aa^	27.96 ± 3.25^aa^	21.39 ± 5.34^aa^	–	84.82 ± 4.87
*P. cyclopium*	17.03 ± 1.23^aa^	13.11 ± 2.01^aa^	24.21 ± 3.45^aa^	13.66 ± 3.28^aa^	–	80.06 ± 5.34
*R. stolonifer*	13.95 ± 0.95^aa^	6.19 ± 0.56^aa^	3.65 ± 1.23^aa^	ND	–	84.63 ± 2.46
*R. solani*	10.88 ± 2.67^aa^	6.21 ± 1.23^aa^	5.94 ± 2.13^aa^	ND	–	30.41 ± 3.26
*S.sclerotiorum*	67.04 ± 4.91^bb^	47.11 ± 3.25	ND	ND	–	34.94 ± 2.35

a ND represents compounds did not show the inhibitory activity against fungi at the concentration of 5 mg/ml.

aa Represents *p < 0.01,* the antifungal activity of extracts were significant weaker compared positive control azoxystrobin, ANOVA with Dunnett`s multiple comparisons test.

bb Represents *p < 0.01,* the antifungal activity of extracts were significant stronger compared positive control azoxystrobin, ANOVA with Dunnett`s multiple comparisons test.

### Cell Viability

**FIGURE 1:**
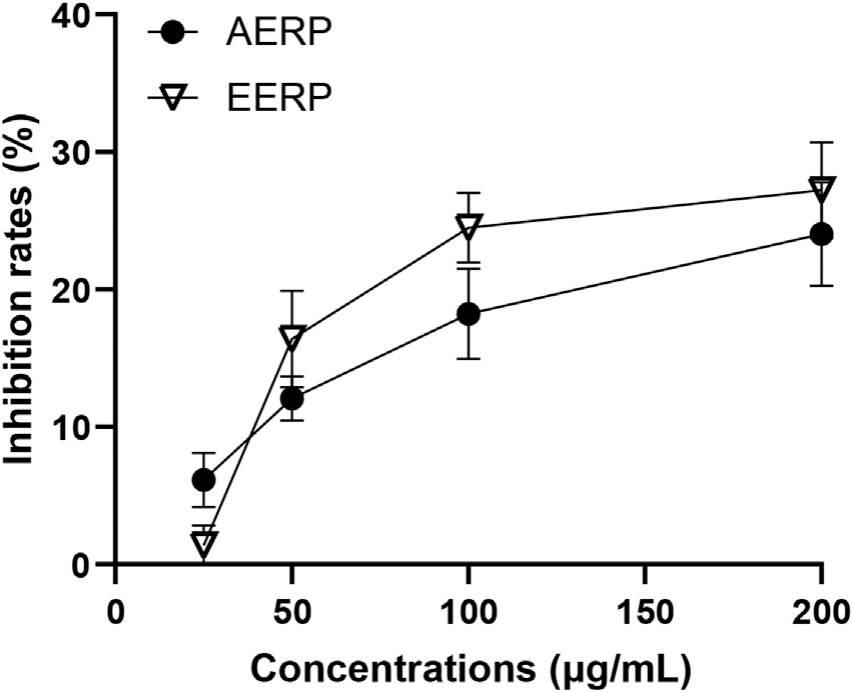
The inhibition rates of the AERP and the EERP against RAW 264.7 cells.

To investigate the anti-inflammatory effects *in vitro*, we first studied the cytotoxicity of the two extracts against RAW 264.7 cells. The results showed that the AERP and EERP presented the weak cytotoxicity in a concentration-dependent manner. At concentrations of 25, 50, 100 and 200 μg/ml, the inhibition rates of the extracts on RAW 264.7 cells were 6.01%, 12.06%, 18.22% and 24.01% for the AERP and 1.28%, 16.39%, 24.50% and 27.21% for the EERP, respectively (Figure 1). The IC_50_ values of the two extracts against RAW 264.7 cells were more than 200 μg/ml. Hence, concentrations of 25–200 μg/ml AERP and EERP were adopted to study their anti-inflammatory activity by measuring the levels of inflammatory cytokines induced by LPS in RAW 264.7 cells.

### Anti-Inflammatory Activity

**FIGURE 2:**
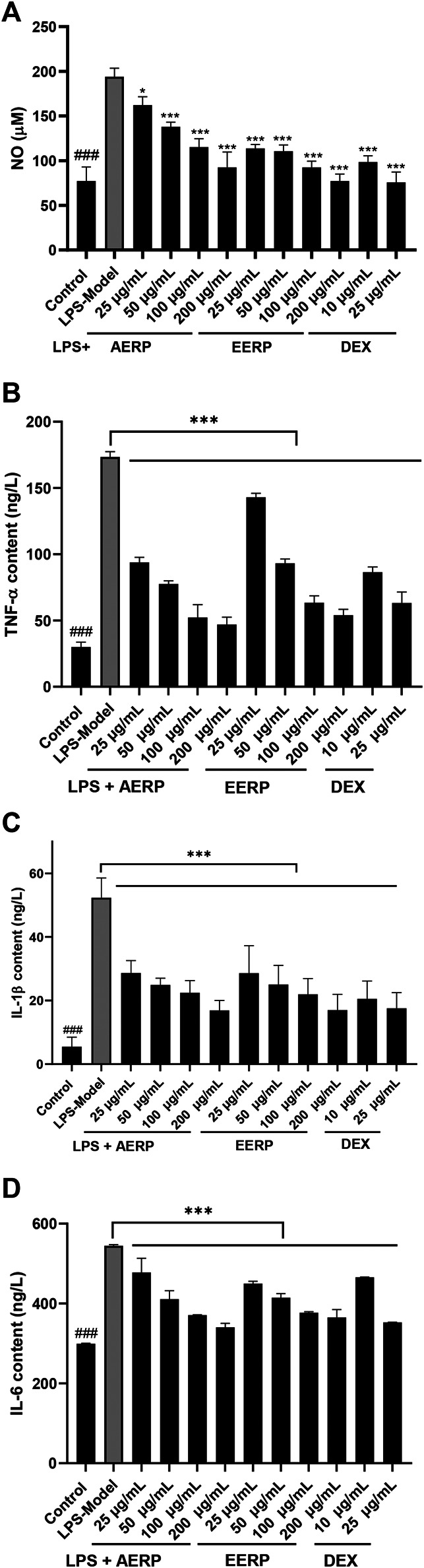
The AERP and the EERP exerted anti-inflammatory effects by inhibiting the production of NO **(A)**, TNF-ɑ **(B)**, IL-1β **(C)** and IL-6 **(D)** induced by LPS in RAW 264.7 cells. (^###^indicates *p* < 0.001 compared with the model group for control by independent *t*-test; ***indicates *p* < 0.001 compared with the model group for the extracts and positive-treatment groups by ANOVA with Dunnett’s multiple comparisons test).

Macrophages play an important role in inflammatory processes and release various cytokines, including NO, IL-1β, IL-6 and TNF-*ɑ*, when stimulated by LPS. NO is a well-known inflammatory cytokine that is released by activated macrophages (Fan et al., 2013; Joo et al., 2014). In this paper, Figure 2A illustrates that at concentrations of 25, 50, 100 and 200 μg/ml, the extracts significantly decreased the content of NO in RAW 264.7 cells stimulated by LPS (*p < 0.01*) with inhibition rates of 27%, 48%, 68%, 87% for the AERP and 69%, 71%, 87% and 100% for the EERP, respectively. Subsequently, the level of TNF*-ɑ* in cells was determined; TNF*-ɑ* is a well-known cytokine that plays an important role in inflammation formation (Genc et al., 2019). The results showed that after LPS treatment, TNF*-ɑ* was stimulated and released. However, when incubated with the different concentrations of the AERP and EERP, the levels of TNF*-ɑ* were markedly decreased, with contents of 41.06, 47.73, 79.02 and 98.71 ng/L for the AERP, and 50.00, 61.74, 97.95 and 156.14 ng/L for the EERP, respectively, compared with those in the control (19.32 ng/L) and LPS model groups (190.38 ng/L) (*p < 0.01*) (Figure 2B).

IL-1β mainly participates in the immune response and tissue repair in response to inflammatory diseases, especially rheumatoid arthritis, and IL-6 has a similar function. The results showed that the AERP and EERP could decrease the production of two other pro-inflammatory cytokines (IL-1β and IL-6, respectively) stimulated by LPS in a dose-dependent manner (*p* < 0.01) (Figures 2C,D).

### Antioxidant Activity

Oxidative stress plays an important role in human health problems, and many natural products with the anti-oxidant activity has been used as safe and effective agents to treat the oxidative stress and its related diseases (Bahadori et al., 2019). In this paper, the antioxidant activity of the two extracts was evaluated in five different assays. The DPPH assay was used to study the reducing power of agents based on an electron transfer reaction. As shown in Table 2, the AERP and EERP showed significant DPPH radical scavenging activity with EC_50_ values of 31 and 25 μg/ml, respectively. The EC_50_ value of the positive control Vc was 7 μg/mL. As one of the free radicals, the superoxide radical could cause cellular damage, and would be contributed to aging and some degenerative diseases (Gutteridge and Halliwell 2000). Meanwhile, the hydroxyl radicals also causes severe the cell death or damage by crossing cell membranes and then react with biomacromolecules (Guo et al., 2017). Further study showed that the extracts also have marked superoxide radical scavenging activity and hydroxyl radical scavenging activity, and the EC_50_ values of the AERP and EERP were 109 and 129 μg/ml and 561 and 668 μg/ml, respectively. For evaluating the antioxidant effect of compounds, the ultimate aim is study the donation of a hydrogen atom to the radical and then the reducing power. Subsequently, the strong reducing power of the AERP and EERP was also demonstrated, and their EC_50_ values were 34 and 40 μg/ml, respectively, while that of the positive control Vc was 28 μg/ml. However, the metal chelating capacity of the two extracts was weak, and the EC_50_ values were all greater than 1,000 μg/ml. This results indicated that the capacity of the two extracts to compete with a chelator to form chelating complexes with iron (II) were weak. The EC_50_ value of the positive control EDTA-2Na was 30 μg/ml.

**TABLE 2:** Antioxidant activities of two extracts and six compounds of *R. przewalskii*.

Extracts	EC_50_ (μg/ml)				
	DPPH radical	Superoxide radical	Hydroxyl radical	Reducing power	Metal chelating
AERP	31 ± 2	109 ± 3	561 ± 7	34 ± 2	>1,000
EERP	25 ± 2	129 ± 4	668 ± 6	40 ± 2	>1,000
Astragalin	22 ± 2	126 ± 6	>1,000	>1,000	>1,000
Kampferol	16 ± 1	54 ± 3	439 ± 5	64 ± 5	>1,000
Quercetin	9 ± 1	24 ± 2	>1,000	12 ± 2	>1,000
Avicularin	39 ± 3	287 ± 10	70 ± 4	42 ± 3	>1,000
Hyperoside	>1,000	174 ± 5	64 ± 3	29 ± 2	>1,000
Azaleatin	37 ± 3	90 ± 2	688 ± 7	27 ± 2	>1,000
Positive	7 ± 1	35 ± 2	161 ± 4	28 ± 1	30 ± 2*

EDTA-2Na was used as positive control for determining metal chelating capacity, the positive control of others assays was Vc.

### Cellular Antioxidant Activity

**FIGURE 3:**
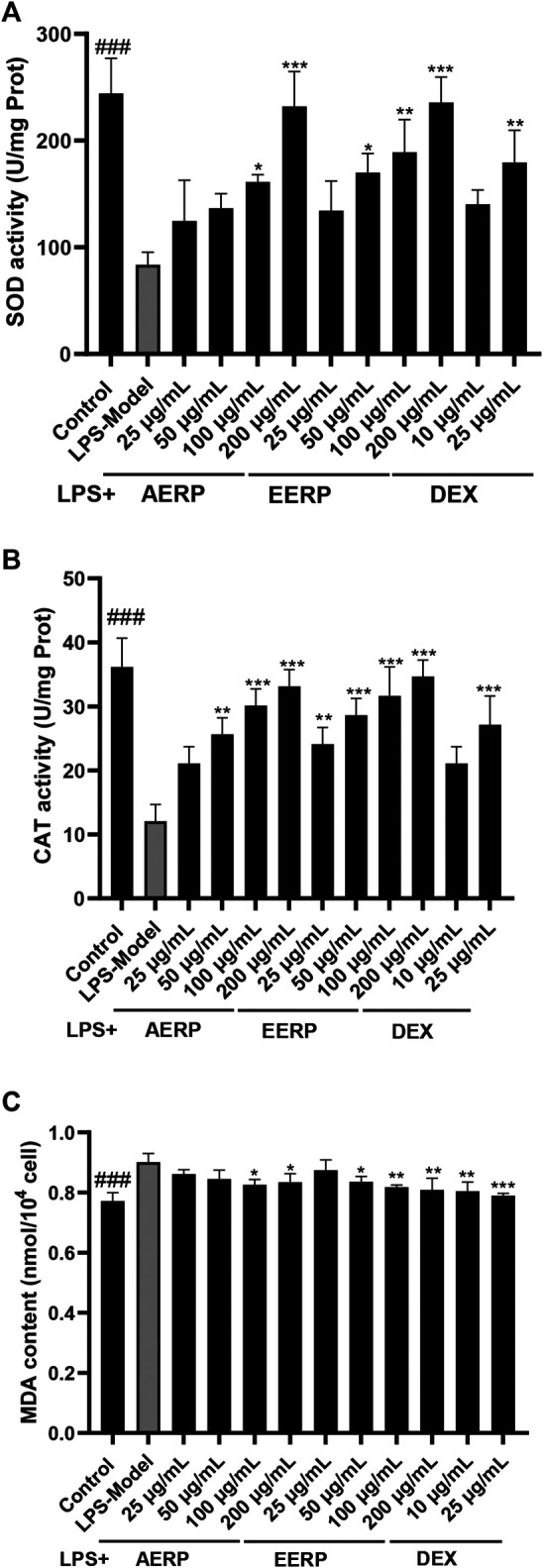
The AERP and the EERP demonstrated cellular antioxidant activity by increasing SOD and CAT activities and decreasing MDA content in RAW 264.7 cells induced by LPS. (^###^ indicates *p* < 0.001 compared with the model group for control by independent *t*-test; ***indicates *p* < 0.001 compared with the model group for extracts and positive-treatment groups by ANOVA with Dunnett’s multiple comparisons test).

Macrophages stimulated by LPS not only result in the production of inflammatory responses and the release of cytokines but also induce the enrichment of superoxide radicals and other radicals (Genc et al., 2019). The above assays proved that the AERP and EERP show marked antioxidant activity and scavenged the radicals *in vitro*. To explain the possible mechanism of the antioxidant activity, the cellular antioxidant activity of the two extracts was investigated by determining the SOD and CAT activities and MDA content induced by LPS in RAW 264.7 cells. Respond to oxidative stress, cells have an antioxidant defense system to prevent cellular damage and increase survival and maintain a balance between free radical production and oxidative stress through enzymatic and non-enzymatic antioxidant defenses (Schieber and Chandel, 2014). SOD and CAT as enzymatic antioxidant defenses provide first-line cellular protection against excess amounts of free radicals (Li et al., 2016; Liao et al., 2014). Currently, most plant antioxidants are investigated the effects by evaluating directly the enzymatic activity of endogenous antioxidants such as SOD and CAT in experimental animals and cells (Kasote et al., 2015). As shown in Figure 3A, the activity of SOD, an important metalloenzyme, was dramatically inhibited after stimulation with LPS (83.69 U/mg prot); however, two extracts (25–200 μg/ml) significantly activated SOD activity in a dose-dependent manner. At concentrations of 100 and 200 μg/ml, the SOD activities were 161.30 and 231.89 U/mg prot for the AERP, and 189.10 and 235.69 U/mg prot for the EERP, respectively (*p* < 0.05). The SOD activity with the positive agent dexamethasone was 140.29 U/mg prot at 10 μg/ml and 179.46 U/mg prot for 25 μg/ml, respectively. After treatment with the two extracts (50–200 μg/ml), CAT activity was also activated in RAW264.6 cells induced by LPS in a dose-dependent manner (Figure 3B). The increase in SOD and CAT activities could help to scavenge free radicals, H_2_O_2_ and other radicals from macrophages induced by LPS. MDA contents are measured as markers of protein and lipid oxidation (Kohen and Nyska, 2002). The MDA levels in cells were used to evaluate the degree of lipid peroxidation. As shown in Figure 3C, the two extracts decreased the production of MDA in cells stimulated by LPS. At concentrations of 25, 50, 100 and 200 μg/ml, the MDA contents were 0.86, 0.85, 0.83 (*p* < 0.05) and 0.83 nM/10^4^ (*p* < 0.05) cells for the AERP and 0.87, 0.84, 0.81 (*p* < 0.01), 0.82 nM/10^4^ (*p* < 0.01) cells for the EERP, respectively, compared with those of the positive agent, 0.80 nM/10^4^ at 10 μg/ml (*p* < 0.01) and 0.79 nM/10^4^ cells at 25 μg/ml (*p* < 0.001). These results indicated that the two extracts demonstrated marked cellular antioxidant activity and that the EERP showed stronger activity than the AERP.

### Chemical Composition of the EERP

#### UPLC-ESI-Q-TOF/MS Analysis

**FIGURE 4:**
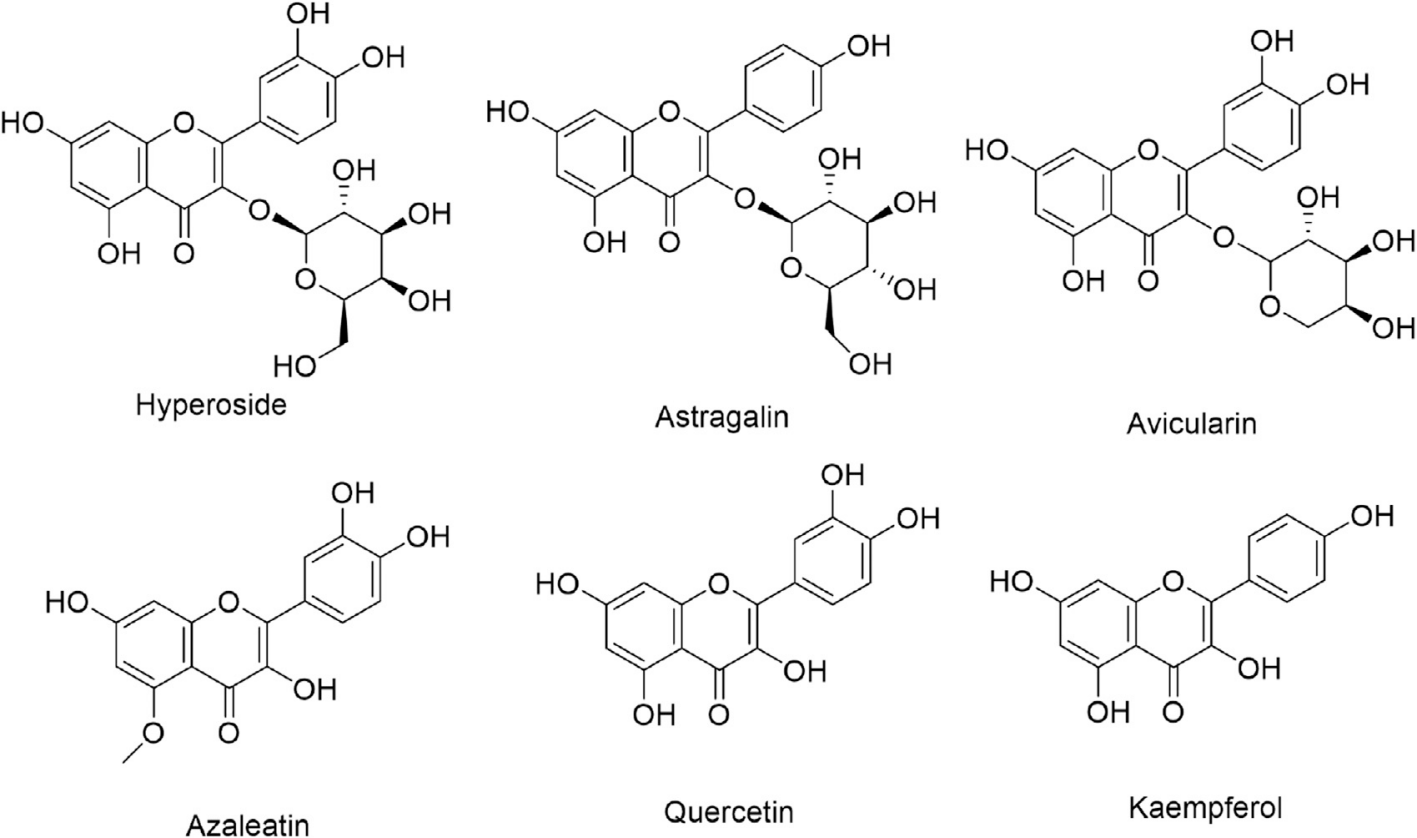
The chemical structure of six identified compounds from R. przewalskii.

**FIGURE 5:**
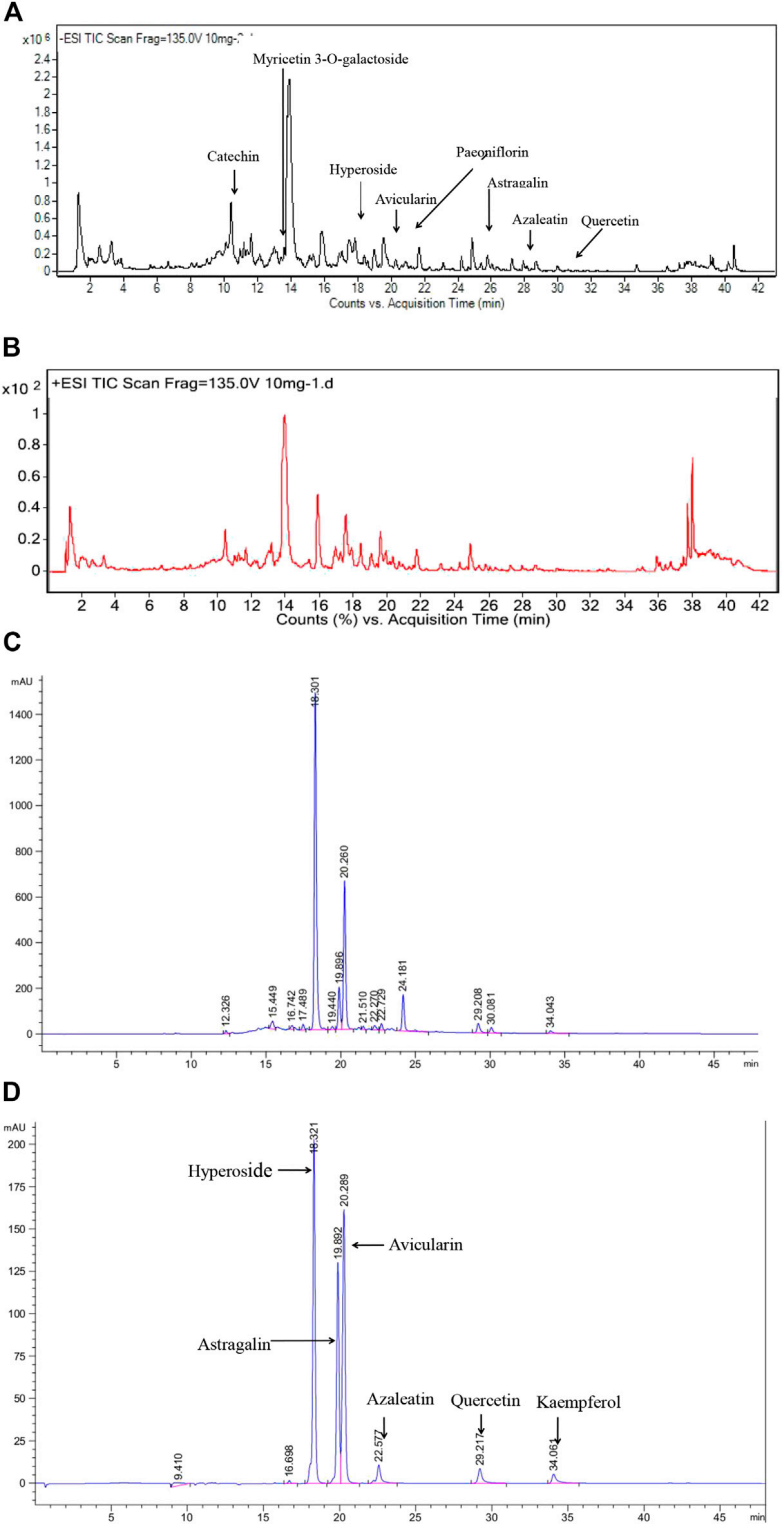
The total ion chromatogram (**(A)** for negative ion, **(B)** for positive ion) and HPLC-UV chromatograms of the EERP **(C)** and the standards **(D)**.

Considering the strong antioxidant activity of the extracts, UPLC-ESI-Q-TOF/MS was used to tentatively identify the composition of the EERP according to its retention time (Rt), pseudomolecular ion formation ([M-H]^–^ and [M-H]^+^). Based on previous research on the *Rhododendron* genus, nine compounds were identified in the negative ion mode, including catechin, myricetin 3-O-galactoside, hyperoside, avicularin, paeoniflorin, astragalin, azaleatin, quercetin and kaempferol (Table 3; Figures 4, 5).

**TABLE 3:** The identified six compounds from *R. przewalskii* ethanol extract.

No	Rt (min)	MS^−^(m/z)	MS^+^ (m/z)	Formula	Proposed compounds	References or HPLC
1	10.30	289.0725	291.0868	C_15_H_14_O_6_	Catechin	TLC
2	12.98	479.0804	–	C_21_H_20_O_13_	Myricetin 3-O-galactoside	Huang et al., 2009
3	17.54	463.0892	465.1029	C_21_H_20_O_12_	Hyperoside	HPLC
4	18.92	433.0783	435.0928	C_20_H_18_O_11_	Avicularin	HPLC
5	20.30	489.1570	491.2409	C_23_H_28_O_11_	Paeoniflorin	TLC
6	24.78	447.0947	449.1095	C_21_H_20_O_11_	Astragalin	HPLC
7	27.27	315.0526	317.0662	C_16_H_12_O_7_	Azaleatin	HPLC
8	28.85	301.0732	303.0511	C_15_H_10_O_7_	Quercetin	HPLC
9	29.95	285.0416	287.0558	C_15_H_10_O_6_	Kampferol	HPLC

#### Quantitative and Qualitative Analysis of the Chemical Composition of the EERP

UPLC was used to study the contents of six compounds in the EERP. Results showed that hyperoside was the most abundant compound, with a yield of 6.03 mg/g fresh material (FM); the other yields were avicularin (2.42 mg/g FM), quercetin (0.27 mg/g FM), azaleatin (0.10 mg/g FM), astragalin (0.08 mg/g FM) and kaempferol (0.08 mg/g FM) (Figures 4, 5) (Table 4). Because the wavelengths of the maximum absorption of catechin and paeoniflorin are 280 nm and 230 nm, respectively, which are different from the absorption wavelengths of the above six compounds, the contents of catechin and paeoniflorin in the extract were checked by TLC.

**TABLE 4:** The content of six compounds in *R. przewalskii* determined by HPLC.

No	Compounds	Rt (min)	Regression line	*R* ^*2*^	Content (mg/g fresh material)
1	Hyperoside	18.32	y = 5,857.6x + 587.14	0.999	6.03
2	Astragalin	19.89	y = 54,546x—19.248	0.999	0.08
3	Avicularin	20.29	y = 6,490.3x—51.66	0.999	2.42
4	Azaleatin	22.58	y = 7,487.8x—35.468	0.999	0.10
5	Quercetin	29.22	y = 5,112.7x—40.898	0.999	0.27
6	Kaempferol	34.06	y = 3,478.5x + 81.073	0.999	0.08

#### The Antioxidant Activity of the Six Compounds

To identify the active compounds from the EERP, we studied the *in vitro* antioxidant activity of the six compounds. The results showed that for DPPH radical scavenging capacity, quercetin presented the strongest activity, with an EC_50_ value of 9 μg/ml, followed by kaempferol (16 μg/ml), astragalin (22 μg/ml), azaleatin (37 μg/ml) and avicularin (39 μg/ml) (Table 1). The activity of hyperosides was weak (>1,000 μg/ml). Quercetin also showed the strongest superoxide radical scavenging activity, with an EC_50_ value of 24 μg/ml, followed by kaempferol (54 μg/ml) and azaleatin (90 μg/ml). The EC_50_ values of other compounds for this activity were all greater than 100 μg/ml. However, in the hydroxyl radical scavenging test, we found that the activity of hyperoside and avicularin was stronger than that of the positive control (161 μg/ml); their EC_50_ values were 64 and 70 μg/ml, respectively. The activities of the other compounds were weak. In addition, except astragalin, the compounds showed marked reducing power. Of these compounds, quercetin and azaleatin exhibited stronger reducing activity, with EC_50_ values of 12 and 27 μg/ml, respectively, compared with that of the positive control (28 μg/ml). However, none of the compounds showed metal chelating activity (Table 2). These results indicated that the compounds presented similar antioxidant activities to those of the extracts*.*

## Discussion

Many *Rhododendron* plants provide health benefits due to their significant bioactivity and have the potential to be utilized in the medicinal and food industries (Kumar et al., 2019). As a dominant evergreen shrub, *Rhododendron przewalskii* is widely distributed on the Qinghai-Tibet Plateau, and the flowers and leaves of *R. przewalskii* are used by local people as folk medicine to treat lung diseases in clinics (Yang et al., 2017). Recently, the essential oils of this species were shown to repel stored-product insects, and this study provided another use for *R. przewalskii* in crop protection (Bai et al., 2019). Due to its long florescence, large flowers, evergreen habit and other characteristics, *R. przewalskii* is used as an ornamental plant in northwestern China (Zheng et al., 2005). To advance the application of this species in the medicinal and horticultural fields, a series of experiments have been carried out to cultivate it through seed propagation (Li et al., 1998a) and vegetative reproduction (Li et al., 1998b). These reports showed that *R. przewalskii* could be widely exploited as an industrial crop. In this paper, we aimed to identify the pharmacological value and active compounds of *R. przewalskii* for potential pharmacological and medicinal uses as an industrial crop and/or as a source for the isolation of active natural products.

Inflammation and oxidative stress are biological responses of the body that occur in many chronic disorders; these responses, when uncontrolled, result in serious injury and disease (Medzhitov, 2008). Many traditional medicines and natural products with anti-inflammatory and antioxidant activities are used as safe and effective agents to treat inflammation, oxidative stress and related diseases (Bahadori et al., 2019). Reports also showed that some plants in the *Rhododendron* genus presented anti-inflammatory and antioxidant activities, such as *R. tomentosum* ssp*. subarcticum* (Black et al., 2011a, b)*, R. arboreum* (Gautam et al., 2020) and *R. ponticum* (Erdemoglu et al., 2008). In this paper, the anti-inflammatory activity of *R. przewalskii* extracts *in vitro* was evaluated for the first time. The results showed that the AERP and EERP presented strong anti-inflammatory activity *in vitro* and could inhibit the inflammatory response by decreasing the production of cytokines such as NO, IL-1β, IL-6 and TNF-*ɑ* in RAW 264.7 cells stimulated by LPS. Further studies found that the two extracts have promising antioxidant activity and that they could interact with free radicals as electron donors; scavenge significant amounts of DPPH radicals, superoxide radicals and hydroxyl radicals; and exhibit reducing power in their antioxidant role. The extracts also markedly increased cellular antioxidant activity by increasing SOD and CAT activities and decreasing MDA content induced by LPS in RAW 264.7 cells. However, the antifungal activity of the two extracts was weak. These results indicated that *R. przewalskii* could be used as a natural antioxidant and anti-inflammatory agent in the pharmaceutical industry. Further mechanisms of their anti-inflammatory and antioxidant activities should be investigated.

Subsequently, UPLC-ESI-Q-TOF/MS was used to tentatively identify the compositions of the extracts, and UPLC-UV was applied to determine the contents of six identified compounds in the extracts. As shown in Figure 4A, approximately 24 compounds from *R. przewalskii* were observed in the total ion chromatogram. However, by comparing the mass spectrometric data of the compounds in this study to the literature related to the *Rhododendron* genus, the natural product library and reference standards, only nine compounds were identified; the other unidentified compounds should be investigated further to identify possible active molecules from *R. przewalskii.* Although the nine identified compounds were found previously in different plants of the *Rhododendron* genus, five compounds, namely, myricetin 3-O-galactoside, paeoniflorin, astragalin, azaleatin and kaempferol, were identified from *R. przewalskii* for the first time*.*

Then, we determined the content of six of the identified compounds. The results showed that *R. przewalskii* contained remarkable amounts of valuable flavonoids. The content of hyperoside (6.03 mg/g FM) in this plant was higher than that in most *Rhododendron* genera, such as *R. anthopogonoides* (Xiao et al., 2018), *R. mariae* (Li et al., 2019) and *R. anthopogonoides* (Li et al., 2015), and even higher than that in *Hypericum perforatum*, which is distributed in China (Yang et al., 2015; Sun et al., 2018) and is widely used to extract hyperoside as an industrial crop. The content of avicularin (2.42 mg/g FM) in *R. przewalskii* was greater than that in *Polygoni avicularis* (Wu et al., 2009; Ban et al., 2019), which was thought to be the main natural source of this compound. Although the quercetin content in *R. przewalskii* (0.27 mg/g FM) was lower than that in *R. groenlandicum* (Mok et al, 2013) and *R. anthopogonoides* (Xiao et al., 2018), it was higher than that in *R. mariae* (Li et al., 2019) and *R. mucronulatum albiflorum* (Mok et al, 2013)*.* The six compounds presented significant antioxidant activity as the extracts did and were thought to be the active compounds in the ethanol extract of *R. przewalskii.*

In view of the significant antioxidant properties of these compounds, *R. przewalskii* could be used as an industrial crop and a potential source for isolating natural antioxidant and anti-inflammatory agents for their comprehensive utilization in the pharmaceutical industry. Because the harvesting time of *Rhododendron* plants affects their phenolic compound content and the antioxidant and anti-inflammatory activities of the compounds (Black et al., 2011a, 2011b), further studies should be performed to investigate the relationships among harvesting time, phenolic content and bioactivity to advance the sustainable use of *R. przewalskii*.

## Conclusion

In this paper, we first studied the antifungal, anti-inflammatory and antioxidant activities of *R. przewalskii* extracts and analyzed their chemical composition. The results showed that *R. przewalskii* extracts presented promising anti-inflammatory and antioxidant activities and that their antifungal activity was weak. The extracts contained high amounts of valuable flavonoids (8.98 mg/g fresh material) and nine compounds were identified. As the well-known antioxidants, they were likely the active compounds contributing to the antioxidant activity of this plant. *R. przewalskii* could be regarded as a potential new source of bioactive ingredients for the pharmaceutical industry. Considering the high yield of flavonoids, more compounds in this plant should be isolated and identified further, and their activities also should be investigated in the future.

## Data Availability

The original contributions presented in the study are included in the article/Supplementary Material, further inquiries can be directed to the corresponding author.

## Abbreviations:

AERP, the aqueous extract of *Rhododendron przewalskii*; CAT, catalase; DEX, dexamethasone; DPPH, 1,1-diphenyl-2-picrylhydrazyl; EDTA, ethylenediaminetetraacetic acid; EERP, the ethanol extract of *R. przewalskii*, FeSO_4_, ferrous sulfate; FeCl_2_, ferrous chloride; IL-1β, interleukin-1β; LPS, lipopolysaccharide; MDA, malondialdehyde; MS, mass spectrometry; NADH-2Na, nicotinamide adenine dinucleotide disodium salt; NBT, nitroblue tetrazolium; NO, nitric oxide; PMS, phenazine methosulfate; SOD, superoxide dismutase; TCA, trichloroacetic acid; TBA, thiobarbituric acid; TLC, thin layer chromatography; TNF-*ɑ*, tumor necrosis factor-*ɑ*; UPLC, ultra-performance liquid chromatography; Vc, ascorbic acid; WST, 2-(2-methoxy-4-nitrophenyl)-3-(4-nitrophenyl)-5-(2,4-disulfophenyl)-2H-tetrazolium and monosodium salt.
