# Supplementary material for: Multiple Biological Activities of Rhododendron przewalskii Maxim. Extracts and UPLC-ESI-Q-TOF/MS Characterization of Their Phytochemical Composition
Source: Front Pharmacol. 2021 Feb 10;12:599778. doi: 10.3389/fphar.2021.599778 (PMC7957927; doi:10.3389/fphar.2021.599778)
Supplement: Supplementary file 1 [file table1.doc]

Table S1. Inhibition zone diameter of tested extracts

| Fungi | AERP | | EERP | | Ketoconazole | Control |
| --- | --- | --- | --- | --- | --- | --- |
|  | 5 mg/mL | 2 mg/mL | 5 mg/mL | 2 mg/mL |  |  |
| *A. niger* | 6 mm | 6 mm | 6 mm | 6 mm | 9.08 mm* | 6 mm |
| *S. cerevisiae* | 6 mm | 6 mm | 6 mm | 6 mm | 11.19 mm* | 6 mm |
| *C. albicans* | 6 mm | 6 mm | 6 mm | 6 mm | 9.34 mm* | 6 mm |

*The concentrations of ketoconazole were 0.025 mg/mL, 0.013 mg/mL and 0.025 mg/mL for the inhibition zone diameter 9.08 mm, 11.19 mm and 9.34 mm, respectively
