# Supplementary material for: Multiple Biological Activities of Rhododendron przewalskii Maxim. Extracts and UPLC-ESI-Q-TOF/MS Characterization of Their Phytochemical Composition
Source: Front Pharmacol. 2021 Feb 10;12:599778. doi: 10.3389/fphar.2021.599778 (PMC7957927; doi:10.3389/fphar.2021.599778)
Supplement: Supplementary file 2 [file table2.doc]

**Cellular antioxidant activity**

The antioxidant activities were investigated by determining the SOD (superoxide dismutase) and CAT (catalase) activities and MDA (malondialdehyde) contents of the RAW 264.7 cells according to the previously described methods (Chou et al., 2013; Wang et al., 2015; Zhang et al., 2021). RAW 264.7 cells (500 μL) at a density of **1×10^4^ cells/well** were incubated in 48-well plates for 24 h, and the cell supernatants were discarded. 500 μL of the AERP and EERP (10-500 μg/mL) were added and cultured for 1 h, and DEX (10 and 25 μg/mL) was used as a positive control. LPS was used to stimulate RAW 264.7 cells for 24 h. Then, the cell supernatants were discarded again, and the cell were digested with trypsin and certrifuged at 8,000g for 10 min at 4 °C, the cell were harvested. After adding into cell lysis buffer, the samples were ultrasonically lysed 30 times (3 s each) on ice with and centrifuged at 8,000g for 10 min at 4 °C again. Finally, the supernatants were collected to determine the SOD and CAT activities and MDA content using the relative assay kits (Solarbio, China). The protein concentrations of each samples were determined using BCA kit (Solarbio, China). Briefly, SOD activity in the supernatant was measured by using nitro blue tetrazolium as a substrate with SOD assay kit. After adding reagents according to the kit to supernatant and then incubating at 37 °C for 40 min, color developing agent was added and kept for 10 min. The absorbance was measured at 560 nm using a Multiskan Go Microplate Spectrophotometer (Thermo Scientific., U.S.A). One unit of SOD activity was defined as the amount of enzyme that inhibited autooxidation by 50% under the given experimental condition and the values were expressed as U/mg prot. CAT activity was determined by using H_2_O_2_ as a substrate with CAT assay kit, and the absorbance was scanned at 240 nm with a Multiskan Go Microplate Spectrophotometer (Thermo Scientific., U.S.A). The concentration of MDA, a reliable marker of lipid peroxidation, was measured using a Multiskan Go Microplate Spectrophotometer at 450, 532 and 600 nm. Three replicates were performed.

**Results**
